# A Mini-Mental State Examination Formula May Help to Distinguish Alzheimer’s Disease from Dementia with Lewy Bodies

**DOI:** 10.3233/JAD-220392

**Published:** 2022-09-27

**Authors:** Tom Ala, Danah Bakir, Srishti Goel, Nida Feller, Albert Botchway, Cindy Womack

**Affiliations:** Dale and Deborah Smith Center for Alzheimer’s Research and Treatment, Southern Illinois University School ofMedicine, Springfield, IL, USA

**Keywords:** Alzheimer’s disease, dementia, Lewy body dementia, memory loss, Mini-Mental State Examination, neurocognitive tests, neuropsychology

## Abstract

**Background:**

Alzheimer’s disease (AD) and dementia with Lewy bodies (DLB) differ in their memory, attention, and visuoconstructional characteristics. The subscales of the well-known Mini-Mental State Examination (MMSE) provide an opportunity to assess these characteristics. Previous research has shown that analysis of the MMSE subscale performance of AD and DLB patients helps to differentiate them.

**Objective:**

Study the MMSE scores of AD and DLB patients to see if the ability of previously reported analyses to differentiate them could be improved. Include other dementia patients for perspective.

**Methods:**

We studied the MMSEs of all patients seen in our clinics during an 18-month period. Different equations were studied, derived from the subscales of Memory (M, 3 points maximum), Attention (A, 5 points maximum), and Pentagon-copying (P, 1 point maximum).

**Results:**

We obtained 400 MMSEs, 136 from AD patients and 24 from DLB patients, scoring range 1–30. The equation P minus M provided the best discrimination between AD and DLB. Using a P-M score = 1 to identify AD, the positive predictive value was 0.97, negative predictive value 0.22, specificity 0.92, and sensitivity 0.43. As a secondary finding, the P-M = 1 equation was also helpful to differentiate AD from Parkinson’s disease dementia.

**Conclusion:**

Considering AD versus DLB in our clinic population, a demented patient who was unable to recall the three memory words on the MMSE but able to copy the intersecting pentagons had a 97% likelihood of having AD. Additional work is needed to improve the sensitivity of the P-M = 1 equation.

## INTRODUCTION

There has been much interest among dementia specialists in using a short cognitive screening exam to help differentiate those with Alzheimer’s disease (AD) from those with dementia with Lewy bodies (DLB) [1–12]. In part due to its ubiquity and simplicity, the Mini-Mental State Examination (MMSE) [13] has been extensively studied for this purpose [8, 10, 14–16].

An emphasis of much of this work has been on the relative neuropsychological differences between AD and DLB, with AD having better attentional and visual processing ability and DLB having better memory [17–19]. For example, Ala et al. [1] was one of the first groups to report the potential usefulness of the MMSE for this purpose, studying AD and DLB patients who had come to autopsy. Using a formula based on the MMSE subscale scores of attention, memory, and pentagon-copying, they reported that the formula distinguished DLB from AD with a sensitivity of 0.82 and a specificity of 0.81.

Other groups have since reported similar differences between DLB and AD using MMSE subscales, particularly the pentagon-copying subscale [3, 5, 6, 10, 11, 14]. For example, Caffara et al. [5] proposed the five-step Qualitative Scoring Pentagon Test (QSPT), reporting that the QSPT had a sensitivity of 70.29% and a specificity of 78.67% to distinguish DLB from AD. Using only the pentagon-copying score with autopsy-confirmed AD and DLB cases, Ala et al. [20] reported that an unacceptable copy was associated with DLB with a sensitivity of 88% and a specificity of 59%.

We report herein our research to further investigate the aforementioned relative neuropsychological differences between AD and DLB, to see if a simple equation could be determined that had improved specificity and/or sensitivity. Continuing the work of others, we focused on manipulating the MMSE subscale scores for Attention (A), Memory (M), and Pentagon-copying (P), ranging from the complicated original Ala formula [1] to simply considering individual subscale scores. For comparison, we scored the patients’ pentagon copies using both the original Folstein single step scoring method [13] and the five-step QSPT method [5].

A secondary objective was to explore whether an equation that was optimal for an AD and DLB cohort would be helpful to distinguish AD or DLB from cognitively impaired patients with other diagnoses. In order to broaden our scope, we included our entire day-to-day clinic population, regardless of level of impairment.

## METHODS

### Study setting

The research was a medical student research project investigating how patients with different neurological conditions completed the MMSE. Four hundred MMSEs acquired from consecutive unique patients who had visited our memory and movement disorder clinics during an approximate 18-month period were reviewed for this study, regardless of diagnosis or reason for visit. The number 400 was chosen arbitrarily, primarily based on the available time for the students. The MMSEs had been routinely administered to almost all new patients and most follow-up patients seen in the two clinics.

### MMSE acquisition

**Fig. 1:**
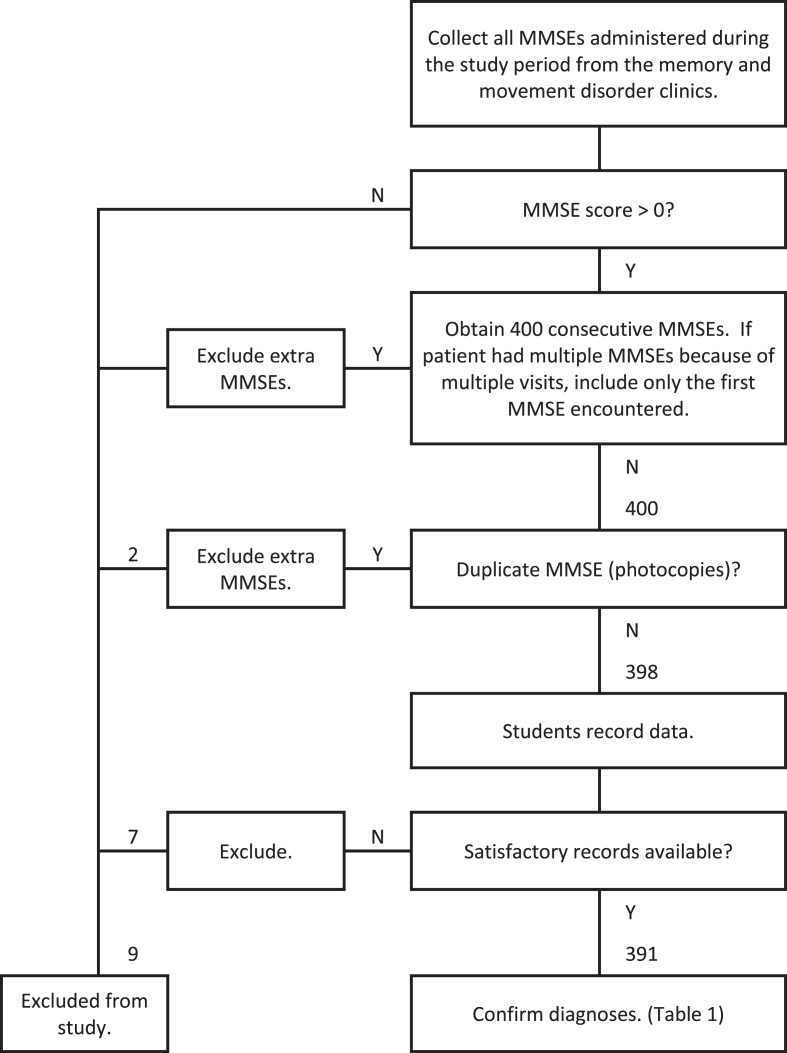
Flow diagram of data acquisition.

The MMSEs were unselected with respect to date or score. If a patient was seen more than once during the study period, only the first MMSE encountered was used. MMSEs obtained from patients who could not complete an MMSE because of visual, hearing, language, orthopedic, or other physical limitations were excluded. Any MMSE score greater than zero was included. Figure 1 shows a flow diagram of how the MMSEs were acquired.

The first task of the students was to obscure the names of the clinicians written on the MMSEs to ensure the clinician’s name did not bias the scoring review of the MMSEs, since one clinician saw mostly movement disorder patients and two saw mostly memory disorder patients. Any diagnostic clues written on the MMSEs were also obscured. After blinding the MMSEs, the students then reviewed each MMSE score for accuracy, rescored if any errors, and recorded the total score, the individual item scores, and the subscale scores on a spreadsheet. Patient demographic details, diagnosis, and medications were also recorded.

The MMSEs were scored according to the original MMSE instructions [13]. By convention, for the Attention and Calculation subscale score, we only scored spelling the word WORLD backwards. Any secondary serial 7 s scoring was not included in our analysis. (In our clinical practice we have found it simpler and more consistent to only use spelling WORLD backwards, especially since many patients have more difficulty with serial 7 s.) The intersecting pentagons copies were scored according to the original instructions [13]: “All 10 angles must be present and 2 must intersect to score 1 point. Tremor and rotation are ignored.” Accordingly, the copies were scored either correct for one point or zero for incorrect.

Since the pentagon copies were an important factor in our study, we also graded them using the five point QSPT scoring method [5]. The QSPT scores the copies using the number of angles, the accuracy of the pentagons’ intersection, the closure of the corners of the pentagons, the correctness of the rotation of the figures, and whether the copy encroaches on the model (closing-in).

### Patient diagnoses

The senior author (TA) reviewed the electronic health records to confirm the best clinical diagnosis for each patient. This was done without knowledge of how the patients answered the individual items of the MMSE. All available clinical data including formal neuropsychological testing results were considered in assigning the best diagnosis. Impairment of his/her social or occupational functioning was a key factor in determining whether a patient was judged to have dementia [21, 22], independent of his/her MMSE score. To be included in the analysis we required each patient to have had at least two visits to our clinics during the study period for diagnosis confirmation, since many with only a single visit had not had a complete work-up.

The patients providing the 400 MMSEs were categorized into the clinical diagnoses listed in Table 1, including the numbers, ages, and MMSE scores of the patients in each category.

**Table 1:** Demographics of patient groups

Patient group	Number	Age (SD)	M/F	MMSE mean (SD)	MMSE range	Comment
Alzheimer’s disease	136	77.8 (11.1)	44/92	20.0 (6.0)	1–30	diagnosis consistent with accepted criteria [21].
Dementia with Lewy bodies	24	78.3 (8.7)	16/8^†^	21.8 (5.1)	10–28	diagnosis consistent with accepted criteria [19].
Parkinson’s disease dementia	18	79.4 (6.6)	9/9	23.3 (5.3)^*^	10–29	diagnosis consistent with accepted criteria [19,33].
Other dementias	26	74.6 (11.3)	16/10^†^	23.4 (5.6)^†^	3–29	patients with other dementias.
Mild cognitive impairment (non-demented)	30	76.9 (7.9)	12/18	25.8 (2.4)^‡^	20–30	diagnosis consistent with accepted criteria [21,22].
Parkinson disease (non-demented)	32	76.5 (9.9)	21/11^†^	28.0 (1.5)^‡^	24–30	diagnosis consistent with accepted criteria [34].
Parkinson disease and mild cognitive impairment (non-demented)	3	81.9 (0.7)	2/1	28.7 (0.6)^*^	28-29	diagnosis consistent with accepted criteria [23].
Mild memory loss	21	70.3 (11.8)^†^	9/12	28.5 (1.2)^†^	26–30	patients with mild findings, not meeting criteria for mild cognitive impairment.
Cognitively intact	29	63.2 (12.5)^‡^	12/17	28.5 (1.6)^†^	24–30	patients judged to have no cognitive impairment.
Other neurological condition, non-demented	11	60.4 (12.3)^‡^	7/4^*^	24.6 (5.6)^*^	14–30	patients with other neurological conditions, non-demented; e.g., stroke, epilepsy, neuropathy.
Demented but uncertain diagnosis	15	74.3 (12.0)	9/6^*^	23.7 (5.2)^*^	10–30	patients with dementia, not clearly meeting criteria for the other categories (unknown diagnosis).
No follow-up	46	68.4 (13.5)^‡^	15/31	26.8 (4.1)^‡^	12–30	patients seen only once in clinic; diagnosis not confirmed.
Excluded	9					excluded from analysis because of duplicate MMSEs or inadequate records.
Total	400

M, male; F, female; MMSE, Mini-Mental State Exam score; SD, standard deviation. ^*^p < 0.05, comparing this group to the Alzheimer’s disease group. ^†^*p* < 0.01, comparing this group to the Alzheimer’s disease group. ^‡^*p* < 0.001, comparing this group to the Alzheimer’s disease group.

The mild cognitive impairment (MCI) category included patients with significant short-term memory impairment but who were not demented, based on whether the patient had impairment of social or occupational functioning [21, 22]. The memory impairment was noted during the clinical exam and corroborated by an informant. A patient did not have to miss all three memory words on the MMSE to be considered MCI. The category of Parkinson’s disease-MCI (PD-MCI) included patients who had both PD and significant memory impairment [23]. Patients who had significant short-term memory impairment and very mild symptoms and/or signs of parkinsonism, not diagnosed with dementia or PD, were categorized as MCI.

### MMSE subscale equations

As our primary purpose was to expand upon the previously published MMSE research comparing AD and DLB patients, we focused on the Attention (A), Memory (M), and Pentagon-copying (P) MMSE subscale scores. In addition, other subscale scores such as orientation and language were also studied. Our intent was to develop a simple, straightforward formula that would be clinically useful. The derivations of the formulae were entirely based on the data; any combination and weighting of the MMSE subscale scores was considered. Table 2 presents the most interesting and potentially useful results.

**Table 2:** Comparing the AD group to the DLB group using different MMSE subscale equations

Subscale equation used to identify AD	Explanation	Was equation satisfied?	Number of AD patients	Number of DLB patients	Odds Ratio	95% confidence interval	PPV	NPV	SENS	SPEC
P-M = 1		yes	59	2	8.43	1.91, 37.28	0.97	0.22	0.43	0.92
		no	77	22
P = 1	only pentagon score	yes	81	8	2.95	1.18, 7.36	0.91	0.23	0.60	0.67
		no	55	16
M = 0	only memory score	yes	106	11	4.18	1.70, 10.27	0.91	0.30	0.78	0.54
		no	30	13
A ≥3	only attention score	yes	95	15	1.39	0.56, 3.43	0.86	0.18	0.70	0.38
		no	41	9
P-M ≥0		yes	116	13	4.91	1.93, 12.47	0.90	0.35	0.85	0.46
		no	20	11
P-M = 0		yes	57	11	0.85	0.36, 2.04	0.84	0.14	0.42	0.54
		no	79	13
M-3P <0		yes	74	7	2.90	1.13, 7.44	0.91	0.22	0.54	0.71
		no	62	17
A-M+P ≥3		yes	96	11	2.84	1.17, 6.86	0.90	0.25	0.71	0.54
		no	40	13
A-5/3M+5P ≥5	original Ala formula [1]	yes	89	9	3.16	1.28, 7.75	0.91	0.24	0.65	0.63
		no	47	15
QSPT P score = 13	scoring the pentagons using the QSPT method^*^	yes	46	6	1.98	0.72, 5.45	0.88	0.21	0.43	0.73
		no	62	16
P = 1	including the same cohort as that scored by the QSPT method^*^	yes	67	7	3.50	1.32, 9.31	0.91	0.27	0.62	0.68
		no	41	15

See Table 1 for AD and DLB patient ages, sex, and MMSE scores. ^*^See text METHODS section MMSE subscale equations for explanation. A, MMSE attention subscale score; AD, Alzheimer’s disease; DLB, dementia with Lewy bodies; MMSE, Mini-Mental State Exam score; NPV, negative predictive value; P, MMSE pentagon-copying subscale score; PPV, positive predictive value; QSPT, Qualitative Scoring Method for the Pentagons Copy Test [5]; SENS, sensitivity; SPEC, specificity.

For the pentagon-copying analysis, our comparison of the original MMSE binary scoring method (correct or incorrect) with the five point QSPT scoring method [5] to differentiate AD from DLB was hindered by a partial loss of data. Inclusion of the QSPT was an afterthought, initiated months after the MMSEs were acquired, and in the interim, the MMSE score sheets from 28 AD and 2 DLB patients were unfortunately lost. This comparison of the smaller cohort is included in Table 2.

Once we determined the best equation for the AD-DLB cohort comparison, we studied how that equation fared in differentiating AD from the other patient groups, as shown in Table 3.

**Table 3:** Comparing the AD and MCI groups to other diagnostic groups using Equation P-M = 1

Comparison groups	Was equation satisfied?	Number of patients in 1st group	Number of patients in 2nd group	Odds Ratio	95% confidence interval	PPV	NPV	SENS	SPEC
AD versus DLB	yes	59	2	8.43	1.91, 37.28	0.97	0.22	0.43	0.92
	no	77	22
AD versus PDD	yes	59	1	13.00	1.69, 100.7	0.98	0.18	0.43	0.94
	no	77	17
AD versus untreated DLB^*^	yes	59	2	4.60	0.99, 21.34	0.97	0.13	0.43	0.86
	no	77	12
AD versus Other dementias group	yes	59	9	1.45	0.60, 3.48	0.87	0.18	0.43	0.65
	no	77	17
AD versus MCI	yes	59	13	1.00	0.45, 2.22	0.82	0.18	0.43	0.57
	no	77	17
MCI versus DLB	yes	13	2	8.41	1.67, 42.40	0.87	0.56	0.43	0.92
	no	17	22

P-M = 1 is the equation based on the MMSE pentagon-copying subscale score minus the MMSE memory subscale score equaling 1. AD, Alzheimer’s disease; DLB, dementia with Lewy bodies; MCI, mild cognitive impairment (non-demented); MMSE, Mini-Mental State Exam score; PDD, Parkinson’s disease dementia; PPV, positive predictive value; NPV, negative predictive value; SENS, sensitivity; SPEC, specificity. ^*^includes only DLB patients who were not treated for parkinsonism.

Since the finding of parkinsonism on exam strongly suggests that a patient more likely has DLB than AD [19], Table 3 includes a subgroup of DLB patients who were not treated with dopaminergic drugs either before or in association with the clinic visits of this study. None of the AD patients were treated for parkinsonism either before or in association with the clinic visits of this study. Whether the AD or DLB patients may have had mild signs of parkinsonism that were not treated was not assessed in this study.

Our study was overseen by the Springfield Committee for Research Involving Human Subjects, which is the institutional review board for Southern Illinois University School of Medicine, in accord with the Helsinki Declaration of 1975.

### Statistics

Descriptive statistics, including means and frequencies, were used to evaluate patient characteristics. Differences in baseline characteristics between the AD and DLB groups and between AD and the other patient groups were analyzed using independent *t*-tests for continuous variables and two-tailed Fisher’s Exact Tests for categorical variables. Significance was determined at the *p* < 0.05 level. 2×2 contingency tables with odds ratios and 95% Woolf approximated confidence intervals were used to compare how the patient groups scored using the different MMSE subscale equations. Positive predictive value (PPV) and negative predictive value (PPV) were calculated using the standard formulae: PPV = TP/(TP + FP) and NPV = TN/(TN+FN), respectively.

## RESULTS

### Demographics

Table 1 presents the demographics of the patients providing the 400 MMSEs in our study. Nine were excluded because of duplicate MMSEs or inadequate records, leaving 391 in the analysis. Since the target groups for this study were the AD and DLB patients, they are listed first. Other groups are also presented to emphasize that this study evaluated the MMSEs acquired from all of the patients who were seen in our clinics and completed MMSEs during the study period. When the demographics of the AD and DLB groups were compared, the AD group had more females (*p* < 0.01); their mean MMSEs and mean ages were not significantly different.

### Subscale equation results

A selection of the most interesting and discriminative equations to compare the subscale scores of the AD and DLB groups is presented in Table 2. The simple equation of Pentagon-copying subscale score minus Memory subscale score (Equation P-M = 1) was found to have the highest PPV (0.97), specificity (0.92), and odds ratio (8.43, 95% confidence interval 1.91, 37.28) to identify AD from our cohort of AD and DLB patients. The various other equations derived from the M, A, and P subscale scores did not yield better results than Equation P-M = 1 to differentiate AD from DLB or to differentiate AD from the other patient groups. A weakness of Equation P-M = 1 was its relatively low sensitivity (0.43). Inclusion of other MMSE subscales like language and orientation was not found to be helpful.

Confirming previous work, the AD group had better attentional and visual processing ability, and the DLB group had better memory [17–19]. Interestingly, as shown in Table 2, just using the individual subscale scores of P or M each resulted in PPVs of 0.91 to differentiate AD from DLB. The specificities of these individual subscale scores were not as high as that for Equation P-M = 1 (0.67 for P, 0.54 for M).

Our study of the MMSE subscales in our AD-DLB cohort did not determine a useful equation for the identification of DLB. The best equation in this regard was P-M<0, which achieved a specificity of 0.85, a weak PPV of 0.35, and a weak sensitivity of 0.46 (data not shown); equation P-M <0 had a good NPV of 0.90 with an odds ratio of 4.91 (95% confidence interval 1.93, 12.47), however.

As shown in Table 2, the PPV of the pentagon-copying test alone to distinguish AD from DLB was less if the more rigorous QSPT method [5] was used to grade the copies (PPV 0.88) instead of the original binary MMSE method (PPV 0.91), although the specificity of the QSPT method was better (0.73 QSPT versus 0.68 original). The odds ratio of the QSPT was also less (1.98 QSPT versus 3.50 original).

As shown in Table 3, if the ten patients who were treated for parkinsonism were excluded from the DLB group, the ability of Equation P-M = 1 to distinguish AD from DLB remained good (PPV 0.97, specificity 0.86).

The ability of Equation P-M = 1 to distinguish AD from the other patient groups with dementia are also included in Table 3. Because of the small numbers of patients with other dementias, such as frontotemporal dementia (FTD, 8 patients) and vascular dementia (7 patients), the patients with other dementias have been combined into the “Other dementias” group. The patients with PD dementia are shown in their own group. The group with MCI (non-demented) is also included for discussion.

For a patient to score 1 using Equation P-M, the patient’s MMSE score could not be 28, 29, or 30. Nine in the AD group and two in the DLB group had scores in that range. Excluding those 11 patients from the analysis did not significantly change the results (data not shown). Twelve in the AD group had MMSE scores less than 10, in contrast to none in the DLB group. Excluding those 12 patients from the analysis did not significantly change the results (data not shown). Only four of the 27 AD patients with MMSE scores <17 fulfilled Equation P-M = 1, as did none of the five DLB patients with scores <17. Excluding those four patients from the analysis did not significantly change the results (data not shown). Whether we considered MMSE score ranges of 1–30, 1–26, 1–27, 10–30, or even 17–27, the PPV, specificity, and sensitivity of Equation P-M to differentiate AD from DLB remained about the same (data not shown).

## DISCUSSION

Our findings again confirm the distinct neuropsychological differences between AD and DLB. The amnestic impairment of AD and the visuoconstructional impairment of DLB clearly help to differentiate them. Considering how our patients with dementia performed on the MMSE, a patient who could copy the pentagons accurately but not remember any of the three memory words had a 97% likelihood to have AD rather than DLB (Equation P-M = 1). We acknowledge the limitation that the relatively low sensitivity of 0.43 of Equation P-M = 1 means that less than half of the AD patients would have been identified, but that fact does not detract from the strong PPV and odds ratio for those whose Equation P-M score was 1.

We emphasize that the benefit of Equation P-M = 1 applied only to those patients who were considered demented, who had a basic dementia workup, and whose differential diagnosis only included AD and DLB. Equation P-M = 1 was useful to identify AD; it was not useful to identify DLB, since its NPV was only 0.22. With the exception of PD dementia, we also did not find it useful to distinguish AD from patients with the other dementias, in part limited by the small numbers of patients with different dementia diagnoses.

If we reduced our AD-DLB cohort to include only those DLB patients who were not treated for parkinsonism (*n* = 14, Table 3), it is remarkable that the results using Equation P-M = 1 to detect AD were almost the same. The PPV and sensitivity remained 0.97 and 0.43, respectively. Since the presence of parkinsonism is included among the criteria for the diagnosis of DLB [19], this finding that Equation P-M = 1 appeared to be independent of parkinsonism strengthens its potential value. Notably, the PPV, sensitivity, and specificity of Equation P-M = 1 to identify AD were even stronger when our cohort of AD and PD dementia patients (excluding DLB patients) were considered (Table 3). We emphasize that the treatment of a subset of the DLB patients with dopaminergic drugs provides only for interesting discussion; we do not promote it as a diagnostic requirement for DLB, since many of the DLB patients manifested only mild parkinsonism (or no parkinsonism) and were not treated.

Another noteworthy detail is prevalence, since prevalence is a factor in the determination of PPV. The prevalence of DLB of 12% (24 of 204 dementia patients with diagnoses, Table 1) in our clinics is somewhat higher than that reported by others, such as Vann Jones and O’Brien (7.5%) [25] and Kane et al. (4.6%) [26]. The most likely explanation for our greater prevalence is the contribution of our movement disorder clinic, since a number of the DLB patients with dementia that had onset less than one year after onset of the parkinsonism [19, 27] were evaluated and followed in our movement disorder clinic. We do not think our enriched prevalence substantially alters our conclusions, nevertheless, since even if our DLB prevalence were halved (e.g., 5.5% instead of 11%), the PPV of Equation P-M = 1 would actually increase to 0.98 to differentiate AD from DLB (assuming identical equation scoring frequencies of the AD and DLB patients).

### Strengths

A strength of our findings is the simplicity of the equation, based on the widely used MMSE, and the fact that we did not select our patients according to severity. All patients seen in our clinics during an 18-month period were included, and the administration and scoring of the MMSEs were done by a variety of clinicians, essentially outside of a research setting. With consideration given to the lack of neuropathological confirmation of the diagnoses of the patients and its relatively low sensitivity, we promote Equation P-M = 1 as a valuable clinical aid but not as a diagnostic criterion.

As another strength, our study included an unselected, non-research, “real world” clinic population, including all patients for whom we obtained an MMSE during the study period. The only patients who were excluded were those who could not score any points on the MMSE or who could not complete the MMSE because of physical reasons. Otherwise, no patients were excluded based on severity or specific diagnosis. We also did not require strict research-level training of those who administered and recorded the MMSEs.

### Limitations

Conversely, the fact that many different providers administered and recorded the MMSEs could be considered a limitation. Residents in training, students, and clinic support staff as well as dementia specialists were involved. Although available, specific directions for MMSE administration were not reviewed before the administration of the MMSE in each case, and all the providers were not specifically trained. This may have resulted in inconsistencies in both administering the test and recording the patients’ responses for the memory and attention subscales.

It would have been interesting if our clinic population had included more patients with other dementias. Having only eight FTD patients is a disappointing limitation of our study in this regard, even though this FTD prevalence of 3.9% (8 of 204, Table 1) is not out of line from population-based reports [28, 29]. Our population also included relatively few vascular dementia and non-Parkinson movement disorder patients, further limiting the generalization of our findings to other dementias

A source of selection bias that potentially weakens our study is the diagnosis of MCI. How many of the MCI patients actually had prodromal AD when they were administered the MMSE? How many may already have converted to AD? How many may actually have had prodromal DLB? How many MCI converted to AD after the MMSE, during the study period? In a retrospective clinical study such as this, when each patient was not systematically queried, examined, followed, and documented, it was very difficult to categorize the patients.

Despite this uncertainty, for this study whether the patient had MCI or AD didn’t make much difference statistically with regards to their performance using Equation P-M = 1 relative to the DLB patients. As shown in Table 3, essentially the same fraction of both groups satisfied the equation (59 of 136 AD, 13 of 30 MCI, both 43%), and the PPVs, NPVs, sensitivities, and specificities of the equation to differentiate them from the DLB group were similar. Since our primary claim is that the equation may be useful to differentiate AD from DLB, whether a patient had late MCI or early AD is therefore not critical. We stress, nevertheless, that we are only advocating its use with patients who have dementia. This study is not addressing prodromal AD, prodromal DLB, or other prodromal dementias.

A valuable follow-up study would be to review subsequent records to see how the MCI patients fared over time. Which, if any, would unquestionably have converted to AD or even to DLB? Furthermore, a follow-up study to assess the accuracy and possible bias in the clinical diagnoses of all of the patients would be very interesting. Ideally, autopsy confirmation would be most helpful.

We acknowledge that our convention of scoring attention (A) by using the spelling of the word WORLD backwards instead of using either serial 7 s or spelling WORLD backwards is a limitation. However, we have found that consistently using WORLD backwards works well for the clinical care of our patients, independent of this study. The tasks are not perfectly equivalent, with variances influenced by education (which we did not systematically assess) and age [30]. Albeit potentially an important detail for future work, for this study we consider the issue of serial 7 s versus WORLD to be of minor importance, since the most useful finding of Equation P-M does not include an A factor.

### Future research

Future research to strengthen the value of the MMSE to identify either AD or DLB could include the addition of clinical features like visual hallucinations, as proposed by Tiraboschi et al. [11], or more complicated visuoconstructional tasks like clock drawing or cube copying, as proposed by Palmqvist et al. [10]. The addition of biomarkers to the MMSE subscale variations should also be further investigated, such as FDG-PET scans, as proposed by Beretta et al. [3], or SPECT scans, as proposed by Hanyu et al. [14] and Yamaguchi et al. [16].

As a final precaution, we emphasize that Equation P-M = 1 showed good retrospective statistical results when our unique clinic patient population was studied. A prospective study is needed, ideally involving other centers with autopsy confirmation of the patients’ diagnoses. Further research could also be done to investigate whether other simple cognitive screening tests like the Montreal Cognitive Assessment [31] or the MiniCog [32] could be useful to distinguish AD from DLB, since both include memory and visuoconstructional tasks. Yamamoto et al. [12], for example, found similar neuropsychological differences between AD and DLB using the Montreal Cognitive Assessment, but their report did not include statistical values such as PPV or specificity.
